# Supplementary material for: Revealing excess protons in the infrared spectrum of liquid water
Source: Sci Rep. 2020 Jul 9;10:11320. doi: 10.1038/s41598-020-68116-w (PMC7347896; doi:10.1038/s41598-020-68116-w)
Supplement: Supplementary file 1 — Supplementary Information. [file 41598_2020_68116_MOESM1_ESM.pdf]

Revealing excess protons in the infrared spectrum of liquid water

Vasily G. Artemov[1,*], **Ece Uykur**[2], **Seulki Roh**[2], **Artem V. Pronin**[2], **Henni Ouerdane**[1], **and Martin Dressel**[2]

[1]Center for Energy Science and Technology, Skolkovo Institute of Science and Technology, 121205 Moscow, Russia
[2]1. Physikalisches Institut, Universität Stuttgart, 70569 Stuttgart, Germany
[*]v.artemov@skoltech.ru

Supplementary Information (SI)

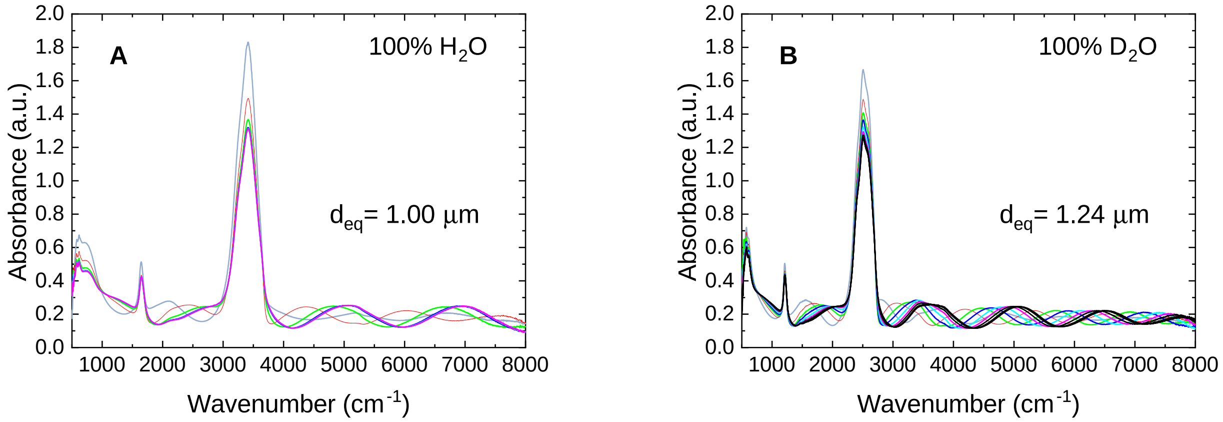

Figure 1. Sets of absorption spectra of water squeezed between two ZnSe windows in infrared frequency region: (A) pure H_2O, (B) pure D_2O. The time interval between the measurements is 30 seconds. The parameter d_{eq} is the effective water layer thickness at equilibrium.

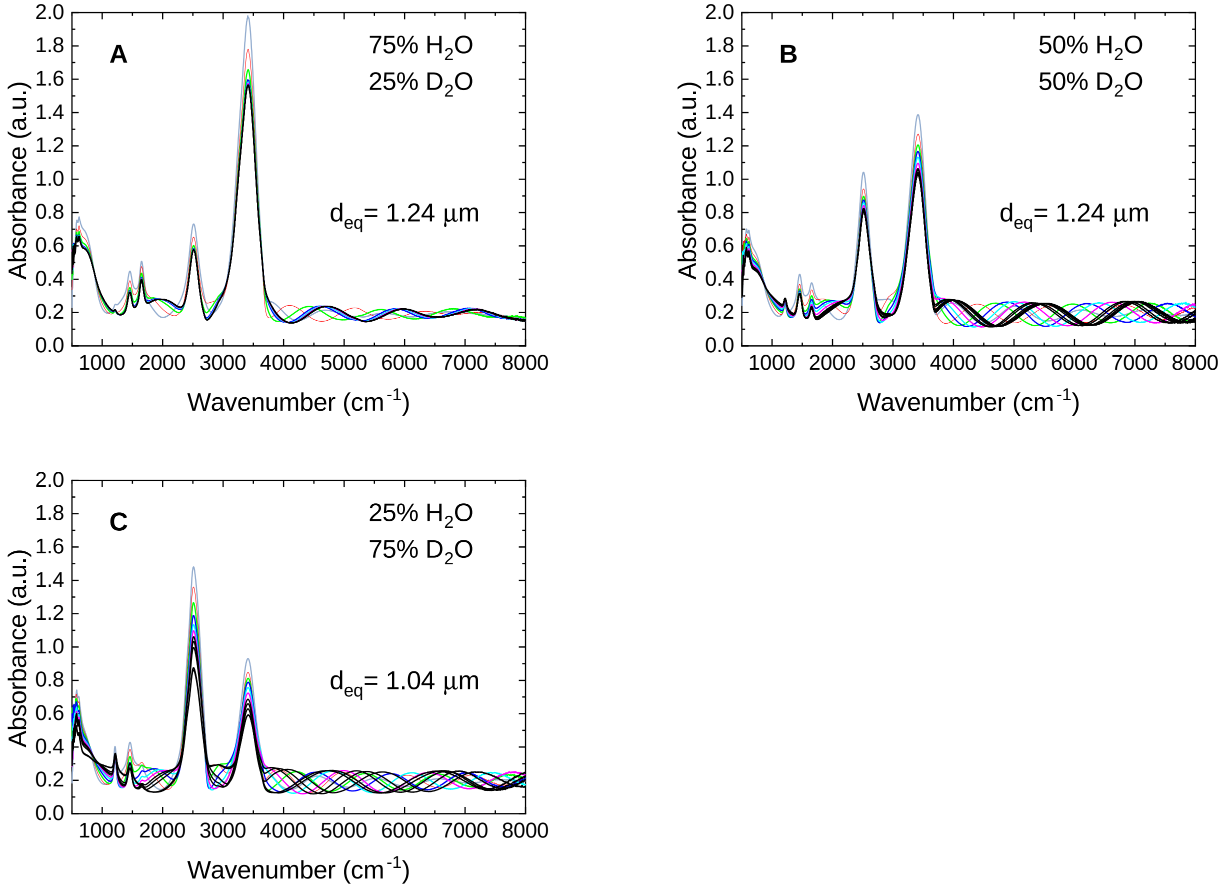

Figure 2. Same as in Fig. 1 for light and heavy water mixtures: (A) 75% H_2O and 25% D_2O, (B) 50% H_2O and 50% D_2O, (e) 25% D_2O and 75% H_2O.

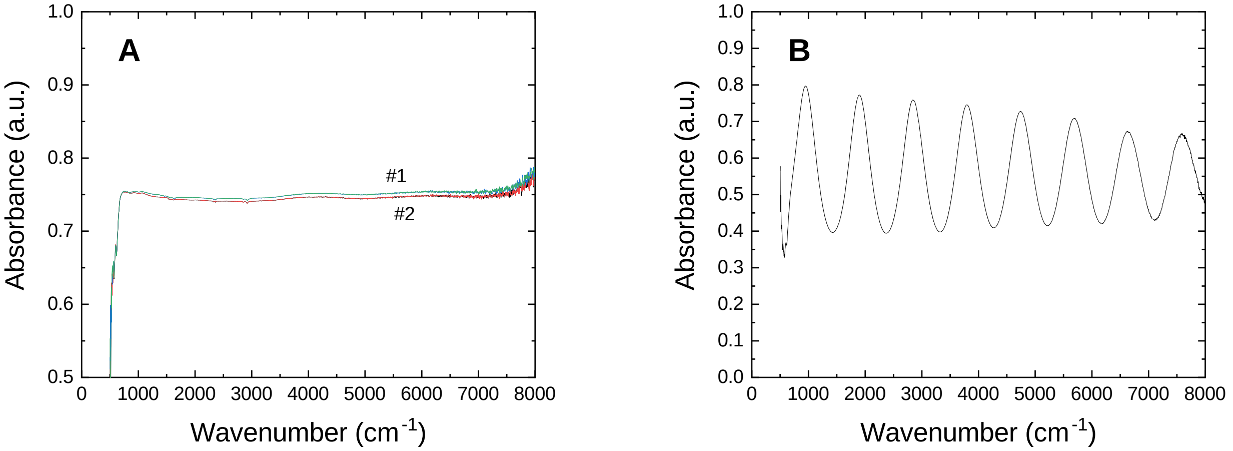

Figure 3. Spectra of optically polished ZnSe windows used for the measurements: (A) separately; (B) two windows together. The thickness of each window is 3.0 ± 0.1 mm.

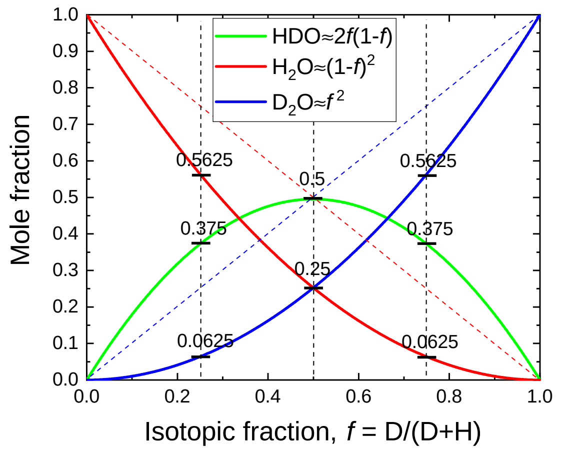

Figure 4. Molar fractions of the components of light and heavy water mixtures as a function of molar isotopic fraction f. The curves are obtained with the equation $K = [\mathrm{HDO}]^2/[\mathrm{H_2O}][\mathrm{D_2O}] \approx 3.85$ and the formulas given in the legend. Numbers near the curves are for $f = 1/4, 1/2, 3/4$ - the molar fractions of the mixtures, whose spectra are shown in Fig. 2.

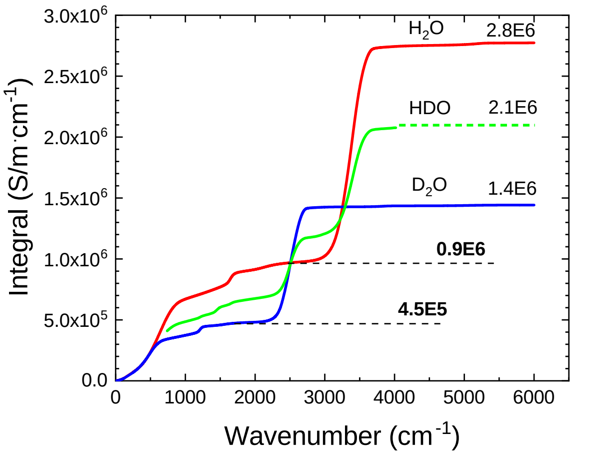

Figure 5. Partial integrals of the spectra shown in Fig. 1, in the main text.

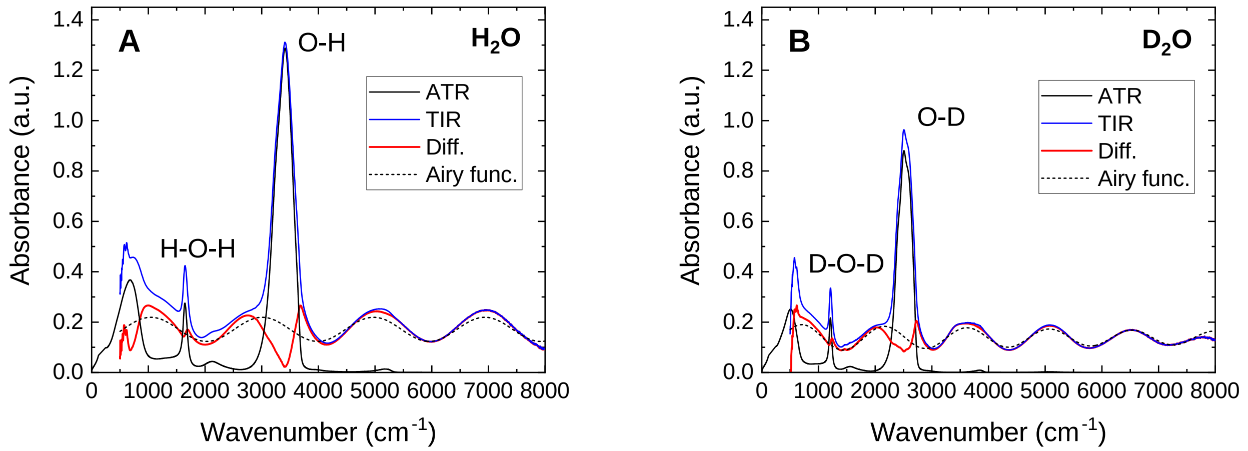

Figure 6. Comparison of ATR (attenuated total reflection - black) and TIR (direct transmission - blue) spectra by subtraction (red). One expects that the difference should be an Airy function, but the characteristic deviations are observed near the stretching and bending modes. The ATR data are from Ref.[1].

References

1. J. J. Max and C. Chapados, J. Chem. Phys. **116**, 4626 (2002).
